# Hospital Expenditure—The Commissariat

**Published:** 1896-10-17

**Authors:** 


### The Institutional Workshop.

### HOSPITAL EXPENDITURE—THE COMMISSARIAT.

### XXX.—GROCERIES.

The articles included among groceries are too numerous to specify at length. They may be classed as follows:—

1. Materials for beverages: Tea, coffee, cocoa, loaf sugar, barley (mineral waters are not included among provisions, but come under the heading of surgery and dispensary).
2. Materials for puddings: Rice, sago, cornflour, and other farinaceous foods; moist sugar, currants, raisins, figs, pippins, and other dried and bottled fruits.
3. Relishes: Salt, pepper, vinegar, mustard, spices, sardines, potted meat, pickles, sauces, jam, &c.
4. Extras for patients, such as jelly and biscuits.
5. Tinned meats: Tongues, pressed beef, brawn, &c.

The list is such a formidable one, and many of the items so expensive, that it is difficult to believe that the total amount expended under this heading works out at, in many cases, a good deal less than the cost of milk. It will readily be seen that the patient's share is far less considerable than that of other members of the staff. His requirements are practically limited to farinaceous foods for puddings and barley water, to seasonings used in cooking, to tea and sugar in hospitals where these necessaries are provided, and to a very varying amount of extras, in the form principally of jelly and biscuits. The cost of tea and sugar for the patient has been already shown to amount to about 3d. a week, or 13s. a year. It would seem that an extra penny a week all round is sufficient to cover the other small items, making a total of 17s. a year in hospitals where all is provided, and 4s. a year in hospitals where tea and sugar are omitted. The cost of the extras is about equalised by the cost of the tea, the patients receiving these particular extras being commonly those on low or milk diet, of which tea does not form a part. The actual average for the patients' groceries at Guy's in the year 1893-4 works out at 15s. per head. It may be therefore claimed that the average of 17s. per head, which we have seen reason to think sufficient, errs, if anything, on the side of liberality.

The following table gives the expenditure on these miscellaneous articles in London and other hospitals for 1893:—

Yearly Expenditure on Groceries.

| | Cost of patients at 17s. per head. £ | s. | d. | Average cost per head of staff. £ | s. | d. |
|---|---|---|---|---|---|---|
| St. George's ... ... | 274 | 0 | 0 | 2 | 0 | 0 |
| Seamen's ... ... | 187 | 0 | 0 | 3 | 0 | 0 |
| Charing Cross ... | 127 | 0 | 0 | 2 | 7 | 0 |
| St. Mary's ... ... | 213 | 0 | 0 | 3 | 0 | 0 |
| St. Thomas's ... | 349 | 0 | 0 | 2 | 0 | 0 |
| Metropolitan ... | 40 | 0 | 0 | 6 | 0 | 0 |
| | Cost of patients at 4s. per head. | | | | | |
| University ... ... | 37 | 0 | 0 | 2 | 0 | 0 |
| Great Northern Central | 13 | 0 | 0 | 2 | 0 | 0 |
| London ... ... | 131 | 0 | 0 | 2 | 16 | 0 |
| London Nursing Home | —— | | | 4 | 0 | 0 |
| Middlesex ... ... | 43 | 0 | 0 | 3 | 11 | 0 |
| Royal Free ... ... | 33 | 0 | 0* | 4 | 0 | 0 |

*Including tea for 10 per cent. of patients.

In provincial institutions the average is much the same, and the cost of the patient does not appear to differ materially in these articles from the London estimate.

Yearly Expenditure on Groceries.

| | Cost of patients at 17s. per head. £ | s. | d. | Average cost per head of staff. £ | s. | d. |
|---|---|---|---|---|---|---|
| Oxford Radcliffe ... | 89 | 0 | 0 | 3 | 2 | 0 |
| Norfolk and Norwich | 114 | 0 | 0 | 3 | 15 | 0 |
| Halifax ... ... | 78 | 0 | 0 | 2 | 16 | 0 |
| Birmingham General | 196 | 0 | 0 | 2 | 6 | 0 |
| Hull Royal Infirmary | 136 | 0 | 0 | 3 | 12 | 0 |
| Leeds General Infirmary | 301 | 0 | 0 | 3 | 8 | 0 |
| Newcastle-on-Tyne | 217 | 0 | 0 | 2 | 7 | 0 |
| Sheffield General Inf. | 151 | 0 | 0 | 3 | 18 | 0 |
| York County ... | 87 | 0 | 0 | 6 | 0 | 0 |
| Liverpool Royal Inf. | 208 | 0 | 0 | 1 | 16 | 0 |
| Aberdeen Royal Inf. | 133 | 0 | 0 | 1 | 12 | 0 |
| Edinburgh Royal Inf. | 586 | 0 | 0 | 2 | 5 | 0 |
| Glasgow Royal Inf. | 461 | 0 | 0 | 2 | 2 | 0 |
| Glasgow Western Inf. | 229 | 0 | 0 | 1 | 10 | 0 |
| Belfast Royal Inf. | 118 | 0 | 0 | 3 | 0 | 0 |
| | Cost of patients at 4s. per head. | | | | | |
| Birmingham Queen's | 22 | 0 | 0 | 3 | 12 | 0* |
| Bristol General ... | 34 | 0 | 0 | 2 | 15 | 0 |

*Including extract of meat.

Deducting from this average for members of the household the sum of £1 per head for tea, coffee, &c., with sugar, there remains over for the cost of all the remaining items a sum not exceeding in many cases 5d. a week each, and in no case amounting to more than 2d. a day. It is well to remember that it is exactly in the use of these articles, which we have indicated above, that any undue extravagance of living shows at once. Everyone who has "kept house" knows what a dangerous leak in the household the grocer's bill may become if little luxuries, even inexpensive ones, are used at all freely. It is, therefore, matter for congratulation to the institutions above named that their published accounts refute in so indisputable a manner the accusation of self-indulgent living which is so frequently and unjustly levelled against members of the hospital staff. It is easy to estimate without much abstruse calculation the length to which 2d. a day will go in luxuries after the cost of puddings and seasonings used in cooking has been deducted.

It should be observed that very small changes in the dietary may modify the grocer's bill, and amply account for the variations noted in the average per head. The use of jam instead of butter, of potted meat in place of cheese, will probably not make the slightest difference in the total cost of the food, but it will relieve the butterman's bill at the expense of the grocer's. Again, the use of tinned meats for supper or breakfast will take a corresponding amount off the cost of meat or bacon, according to circumstances. A good housekeeper keeps a clear idea in her own head of the relative cost of such exchanges, and, moreover, of their relative food value. A dish of tinned salmon or lobster may seem an inexpensive variety as a supper dish, but is an extravagant substitute for plainer fare when regarded as an evening meal for tired and hungry workers.

Co-operative stores have done much to equalise the prices in institutions for most articles of grocery. In London, hospitals often purchase from these direct.

In other places, they need only place the price list of one of the larger stores in the hands of the local tradesmen and may be certain there will be no refusal to supply all that is required at the store price. Yet, in spite of this standard book of reference, prices do vary widely for certain items the cost of which has been ascertained in many London and provincial institutions. The widest variations are in the price of tea, coffee, and cocoa. The latter is comparatively little used, and as hardly two institutions use the same kind it is not worth while to quote prices.

For coffee almost every London hospital pays at the rate of 1s. 4d. or 1s. 3½d. per pound. There are one or two exceptions, where it is obtained for 1s. 1½d. In provincial institutions the price rises to 1s. 5d. at Leeds, 1s. 6d. at Liverpool, 1s. 7d. at Wolverhampton, and to 1s. 9d. at Glasgow. Common prices are from 1s. 1½d. to 1s. 3d. At Brighton the price falls to 11d. per pound, and at Hull to 10d. per pound.

The difference in price as regards tea is much more strongly marked. In London the range of prices before us is from 2s. 4d. to 1s. 4d. (officers' tea), and from 1s. 7d. to 1s. (patients' tea). In two hospitals the price paid is 1s. 7d., in two it is 1s. 6d., in two 1s. 5d., in two 1s. 4d. Other prices are 1s. 3½d., 1s. 2½d., 1s. 2d. Curious anomalies may be observed; whereas in one hospital 1s. 4d. is considered too high a price for any but officials (the patients being provided with something cheaper), in another the lower price for the patients' tea is 1s. 6d., and in others, all the household together are provided at the rate of 1s. 2d. per pound.

Outside London the variation is even greater. Officers' tea costs from 2s. 6d. to 1s. 6d. per pound, patients' tea from 2s. to 10½d. per pound. At Hull and Norwich the price is 1s. 8d.; at Liverpool and Sheffield (where 2s. and 2s. 6d. per pound is paid for officers' tea) the patients' tea costs 1s. 6d.; at Leeds 1s. 5d.; at Birmingham, Oxford, Newcastle-on-Tyne, Bradford, and Wolverhampton the price is 1s. 4d., or nearly so. In some other places as little as from 1s. 1½d. to 10½d. per pound is paid. In Scotch hospitals the prices are: Kilmarnock, 1s. 8d.; Edinburgh, 1s. 6d. (officers' tea, 2s.); Glasgow Western, 1s. 6d.; Ayr, 1s. 5d.; Glasgow Royal, 1s 2d.

In one large London infirmary as little as 6d. per pound is paid for the patients' tea, and considering that the duty alone amounts to 3d. per pound, we think most people will agree that this price marks the existence of a serious scandal.

It is quite clear that widely different views are held by the authorities with regard to the quality of tea deemed suitable for the requirements of the sick poor. Whether the patient gets tea fit to drink is not, however, a question which can be decided off-hand by the price paid. Very probably the local tradesman may supply tea at 1s. 8d. and 2s per lb., vastly inferior to what could be had for 1s. 2d. or 1s. 4d. in bulk direct from the dealer. The advantages of procuring tea in the chest or box from regular tea merchants when large numbers are to be provided, are so obvious that it is remarkable that so many hospitals still cling to the family grocer for this important item in everyone's comfort. In one or two instances where a low price is paid, and the tea is procured from this source, the result is unspeakably bad. The secretary of a large provincial hospital himself informed the writer that the tea brewed for the patients' use had all the appearance of being infused from straw. It was entirely undrinkable by anyone not a patient, and was commonly rejected by those for whom it was provided. The price paid for this trash was 10½d. per lb.

An elementary knowledge of the tea trade would satisfy any manager that genuine tea is not to be obtained even in bulk under 1s. 2d. per lb., and that rarely under 1s. 4d. per lb. can it be guaranteed of really sound quality and fit for household use. We would urge once again on all those in authority the wisdom and dignity of maintaining one standard of quality, and that a high one, with respect to the food of every inmate of the institution.

